# Chemokine-like Receptor 1 in Brain of Spontaneously Hypertensive Rats Mediates Systemic Hypertension

**DOI:** 10.3390/ijms222111812

**Published:** 2021-10-30

**Authors:** Atsunori Yamamoto, Kosuke Otani, Muneyoshi Okada, Hideyuki Yamawaki

**Affiliations:** Laboratory of Veterinary Pharmacology, School of Veterinary Medicine, Kitasato University, Higashi 23 Bancho 35-1, Towada, Aomori 034-8628, Japan; dv20006@st.kitasato-u.ac.jp (A.Y.); otani@vmas.kitasato-u.ac.jp (K.O.); mokada@vmas.kitasato-u.ac.jp (M.O.)

**Keywords:** adipocytokine, chemerin, CMKLR1, PVN, hypertension

## Abstract

Adipocytokine chemerin is a biologically active molecule secreted from adipose tissue. Chemerin elicits a variety of functions via chemokine-like receptor 1 (CMKLR1). The cardiovascular center in brain that regulates blood pressure (BP) is involved in pathophysiology of systemic hypertension. Thus, we explored the roles of brain chemerin/CMKLR1 on regulation of BP in spontaneously hypertensive rats (SHR). For this aim, we examined effects of intracerebroventricular (i.c.v.) injection of CMKLR1 small interfering (si)RNA on both systemic BP as measured by tail cuff system and protein expression in paraventricular nucleus (PVN) of SHR as determined by Western blotting. We also examined both central and peripheral protein expression of chemerin by Western blotting. Systolic BP of SHR but not normotensive Wistar Kyoto rats (WKY) was decreased by CMKLR1 siRNA. The decrease of BP by CMKLR1 siRNA persisted for 3 days. Protein expression of CMKLR1 in PVN of SHR tended to be increased compared with WKY, which was suppressed by CMKLR1 siRNA. Protein expression of chemerin in brain, peripheral plasma, and adipose tissue was not different between WKY and SHR. In summary, we for the first time revealed that the increased protein expression of CMKLR1 in PVN is at least partly responsible for systemic hypertension in SHR.

## 1. Introduction

Adipocytokines are biologically active molecules secreted from adipose tissue [1]. Chemerin encoded by retinoic acid receptor responder protein 2/tazarotene-induced gen 2 is one of the recently discovered adipocytokines [2]. The carboxyl-terminal of prochemerin that is an immature form is cleaved by serine proteases, which produces a mature form of active chemerin [2,3]. Chemerin is highly expressed in adipose tissue, while it is also expressed in liver, lung, and brain [4]. A variety of functions of chemerin have been so far identified (e.g., chemotaxis for immune cells, adipocyte differentiation, and inhibition of cancer growth) [5,6,7]. 

Chemokine-like receptor 1 (CMKLR1), chemokine (CC motif) receptor-like 2 (CCRL2), and G protein-receptor 1 (GPR1) were identified as the receptors for chemerin [2]. CMKLR1 is expressed in immune cells, adipose tissue, lung, heart/blood vessel, and central nervous system [4,8,9]. We have previously demonstrated that chemerin stimulated the proliferation and migration via CMKLR1 in rat vascular smooth muscle cell and that systolic blood pressure in mice was increased by the long-term intraperitoneal administration with chemerin [10]. Additionally, it was reported that the increased expression of chemerin and CMKLR1 in thoracic aorta of obese rats was partly related to the development of systemic hypertension [11]. Taken together, it is suggested that chemerin/CMKLR1 is involved in pathogenesis of hypertension development through acting on reactivity and structural remodeling of peripheral vasculature. On the other hand, CCRL2 expressed in lung and brain did not induce any signal transduction [12]. GPR1 expressed in skeletal muscle and adipose tissue was reported to be related to glucose homeostasis [13].

The cardiovascular center in the brain region regulates blood pressure by controlling cardiac output and vascular reactivity [14]. It locates in the rostral ventrolateral medulla (RVLM) [14,15]. Subfornical organ, organum vasculosum lamina terminalis, and paraventricular nucleus (PVN) are other important central nuclei regulating blood pressure, which affect the functions of peripheral organs via intermediolateral nucleus through projecting to RVLM [14,15,16]. Particularly, PVN is a crucial region that regulates blood pressure through the secretion of vasopressin and/or the activation of sympathetic nerve activity (SNA) [17,18].

Recently, it has been reported that the cardiovascular center is involved in pathophysiology of systemic hypertension. For example, the expression of angiotensin II type 1 receptor was increased in PVN of obese rats, which led to an increase in blood pressure through sympathoexcitation [19]. Moreover, it was reported that a receptor for inhibitory neurotransmitter (γ-aminobutyric acid: GABA) was decreased in PVN of spontaneously hypertensive rats (SHR), resulting in increased blood pressure [20]. Further, leptin, an adipocytokine, that crosses blood brain barrier and affects central nervous system, induced hypertension through sympathoexcitation [21,22]. Recently, we have revealed that intracerebroventricular (i.c.v.) injection of chemerin-9, an active fragment of chemerin, to normal Wistar rats increased blood pressure via CMKLR1 [23]. However, the roles of chemerin/CMKLR1 in brain of SHR on blood pressure remain to be elucidated. In order to explore them, we first examined protein expression of CMKLR1 in brain of SHR. Next, we investigated the effects of i.c.v. injection of CMKLR1 small interfering (si)RNA on systemic blood pressure in SHR.

## 2. Results

### 2.1. Protein Expression of CMKLR1 around Brain Ventricles of Wistar Kyoto Rats (WKY) and SHR

**Figure 1:**
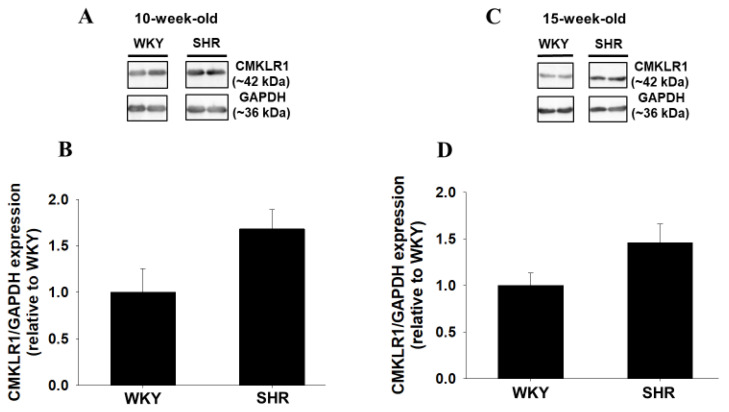
Protein expression of chemokine-like receptor 1 (CMKLR1) around brain ventricles of Wistar Kyoto rats (WKY) and spontaneously hypertensive rats (SHR). Protein expression of CMKLR1 around brain ventricles of male WKY and SHR ((**A**,**B**):10-week-old, (**C**,**D**): 15-week-old) was determined by Western blotting. Representative blots were shown (**A**,**C**). The normalized CMKLR1 expression relative to glyceraldehyde 3-phosphate dehydrogenase (GAPDH) expression was shown as relative to WKY. Data were shown as mean + standard error of the mean (SEM) in bar graph ((**B**): *n* = 4, (**D**): WKY; *n* = 5, SHR; *n* = 4).

Protein expression of CMKLR1 around brain ventricles was compared between normotensive WKY and SHR. Protein expression of CMKLR1 in SHR (10- and 15-week-old) was increased compared with age-matched WKY (Figure 1A,B: *n* = 4, Figure 1C,D: WKY; *n* = 5, SHR; *n* = 4).

### 2.2. Effects of CMKLR1 siRNA on Systolic Blood Pressure and Heart Rate in WKY and SHR

**Figure 2:**
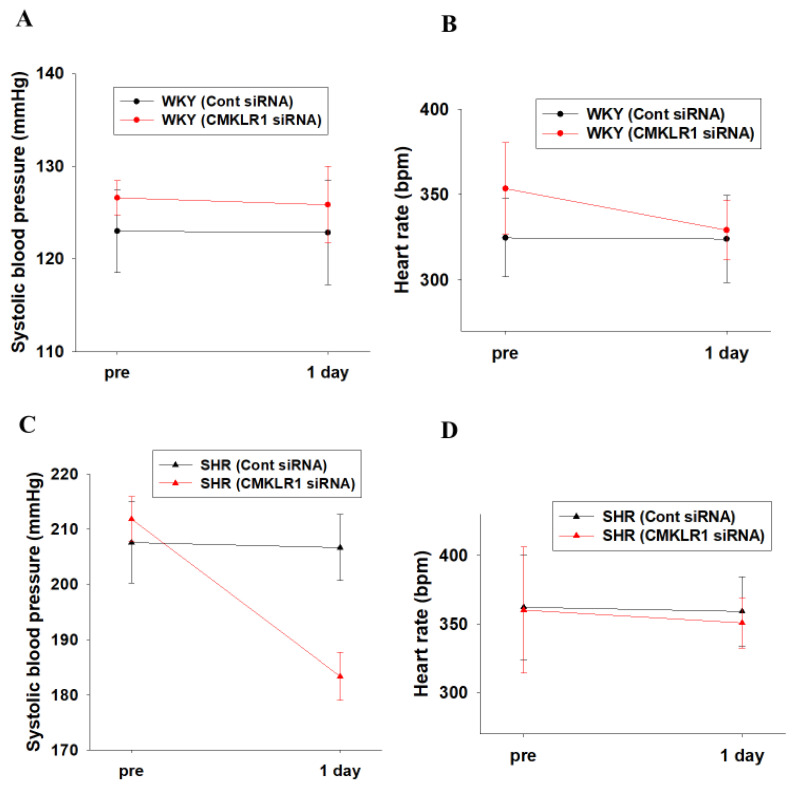
Effects of CMKLR1 small interfering (si)RNA on systolic blood pressure and heart rate in WKY and SHR. Control (Cont) siRNA [0.04 nmol/head, intracerebroventricular (i.c.v.)] or CMKLR1 siRNA (0.04 nmol/head, i.c.v.) was administered to male WKY and SHR for 1 day. Systolic blood pressure (**A**,**C**) and heart rate (**B**,**D**) were measured by tail cuff system. Data were shown as mean ± SEM (*n* = 4). Pre: before administration of siRNA. 1 day: 1 day after administration of siRNA.

Control (Cont) siRNA (0.04 nmol/head, i.c.v.) or CMKLR1 siRNA (0.04 nmol/head, i.c.v.) was administered to WKY and SHR (15–16-week-old) for 1 day. Systolic blood pressure of CMKLR1 siRNA-treated SHR was decreased compared with Cont siRNA-treated SHR (*n* = 4, *p* = 0.057, Figure 2C), while that of CMKLR1 siRNA-treated WKY did not change compared with Cont siRNA-treated WKY (*n* = 4, Figure 2A). Systolic blood pressure of CMKLR1 siRNA-treated SHR at 1 day was decreased compared with pre [Δsystolic blood pressure: −28.5 mmHg in SHR (CMKLR1 siRNA), *n* = 4, Figure 2C], while that of Cont siRNA-treated SHR at 1 day did not change [Δsystolic blood pressure: −0.9 mmHg in SHR (Cont siRNA), *n* = 4, Figure 2C]. On the other hand, heart rate of CMKLR1 siRNA-treated SHR or WKY did not change compared with Cont siRNA-treated SHR or WKY [Δheart rate: −0.7 bpm in WKY (Cont siRNA), −24.5 bpm in WKY (CMKLR1 siRNA), −3.1 bpm in SHR (Cont siRNA), −9.3 mmHg in SHR (CMKLR1 siRNA), *n* = 4, Figure 2B,D].

### 2.3. Short-Term Effects of CMKLR1 siRNA on Systolic Blood Pressure and Heart Rate in SHR

**Figure 3:**
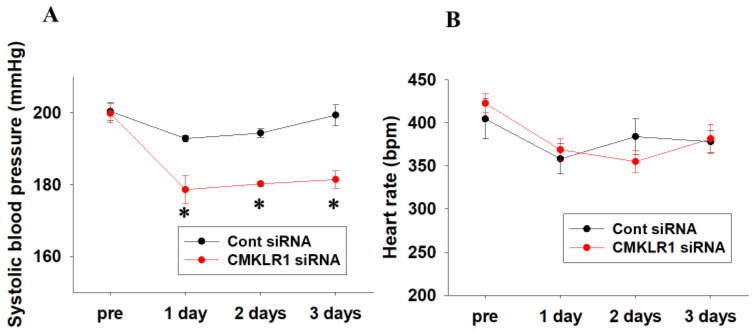
Short-term effects of CMKLR1 siRNA on systolic blood pressure and heart rate in SHR. Cont siRNA (0.04 nmol/head, i.c.v.) or CMKLR1 siRNA (0.04 nmol/head, i.c.v.) was administered to male SHR for 3 days. Systolic blood pressure (**A**) and heart rate (**B**) were measured by tail cuff system. Data were shown as mean ± SEM (Cont siRNA; *n* = 4, CMKLR1 siRNA; *n* = 5). Pre: before administration of siRNA. 1–3 days: 1–3 days after administration of siRNA. * *p* < 0.05 vs. Cont siRNA.

Cont siRNA or CMKLR1 siRNA was next administered to SHR (15–17-week-old) for 3 days. Systolic blood pressure of CMKLR1 siRNA-treated SHR was significantly decreased compared with Cont siRNA-treated SHR, which was sustained for 3 days (Cont siRNA; *n* = 4, CMKLR1 siRNA; *n* = 5, *p* < 0.05 vs. Cont siRNA, Figure 3A). Systolic blood pressure of CMKLR1 siRNA-treated SHR at 1–3 days was decreased compared with pre [Δsystolic blood pressure: 1 day; −21.2 mmHg, 2 days; −19.6 mmHg, 3 days; −18.4 mmHg in CMKLR1 siRNA, *n* = 5, Figure 3A], while that of Cont siRNA-treated SHR was slightly decreased at 1 day and returned to base line at 3 days [∆systolic blood pressure: 1 day; −7.5 mmHg, 2 days; −6.0 mmHg, 3 days; −1.0 mmHg in Cont siRNA, *n* = 4, Figure 3A]. On the other hand, heart rate of CMKLR1 siRNA-treated SHR did not change compared with Cont siRNA-treated SHR (Cont siRNA; *n* = 4, CMKLR1 siRNA; *n* = 5, Figure 3B).

### 2.4. Effects of CMKLR1 siRNA on Protein Expression of CMKLR1 in PVN

**Figure 4:**
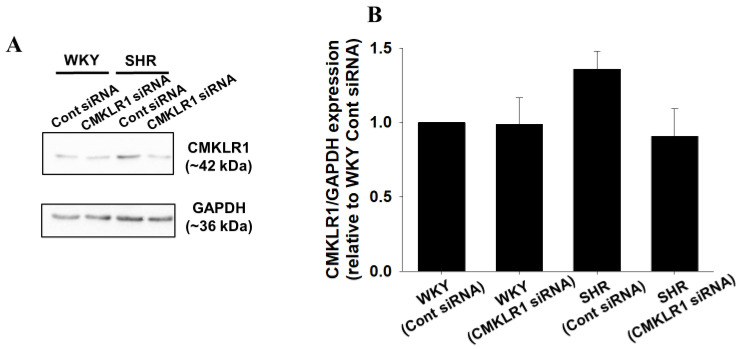
Effects of CMKLR1 siRNA on protein expression of CMKLR1 in paraventricular nucleus (PVN). Cont siRNA (0.04 nmol/head, i.c.v.) or CMKLR1 siRNA (0.04 nmol/head, i.c.v.) was administered to male WKY or SHR. One day later, protein expression of CMKLR1 in PVN was determined by Western blotting. Representative blots were shown (**A**). The normalized CMKLR1 expression relative to GAPDH expression was shown as relative to WKY (Cont siRNA). Data were shown as mean ± SEM in bar graph ((**B**): *n* = 4).

PVN is a crucial region that regulates blood pressure through SNA. Thus, we compared CMKLR1 protein level in PVN between WKY and SHR. Protein expression of CMKLR1 in PVN of Cont siRNA-treated SHR tended to be increased compared with Cont siRNA-treated WKY, which was decreased by CMKLR1 siRNA. On the other hand, protein expression of CMKLR1 in CMKLR1 siRNA-treated WKY did not change compared with Cont siRNA-treated WKY (*n* = 4, Figure 4).

### 2.5. Protein Expression of Chemerin around Brain Ventricles of WKY and SHR

**Figure 5:**
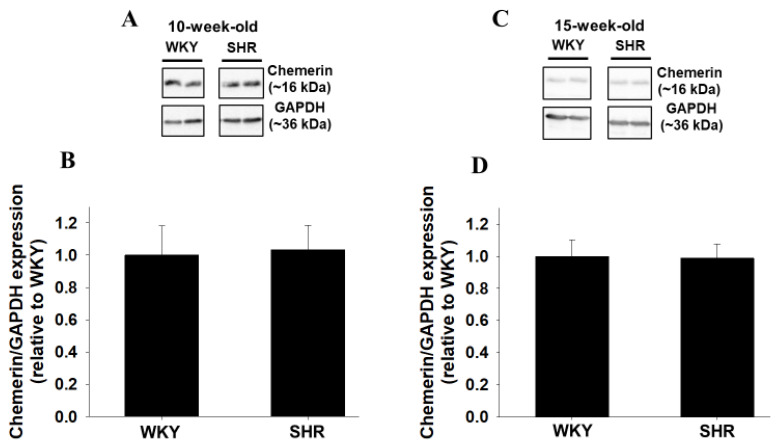
Protein expression of chemerin around brain ventricles of WKY and SHR. Protein expression of chemerin around brain ventricles of male WKY and SHR ((**A**,**B**):10-week-old, (**C**,**D**): 15-week-old) was determined by Western blotting. Representative blots were shown (**A**,**C**). The normalized chemerin expression relative to GAPDH expression was shown as relative to WKY. Data were shown as mean + SEM in bar graph ((**B**): *n* = 4, (**D**): WKY; *n* = 5, SHR; *n* = 4).

Protein expression of chemerin around brain ventricles was compared between WKY and SHR. Protein expression of chemerin in SHR (10- and 15-week-old) did not change compared with age-matched WKY (Figure 5A,B: *n* = 4, Figure 5C,D: WKY; *n* = 5, SHR; *n* = 4).

### 2.6. Protein Expression of Peripheral Chemerin in WKY and SHR

**Figure 6:**
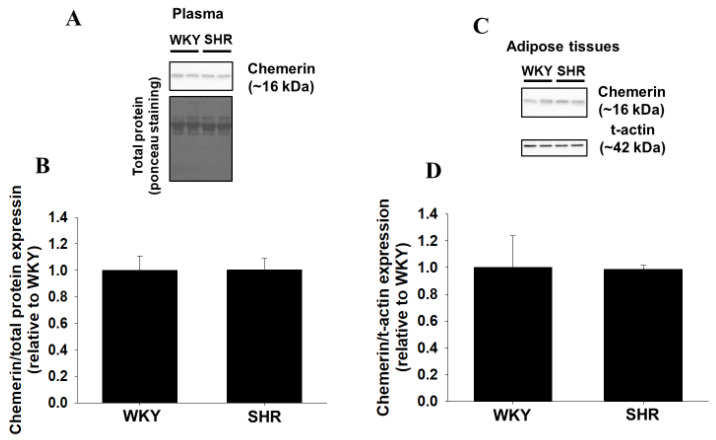
Protein expression of chemerin in plasma and adipose tissues of WKY and SHR. Protein expression of chemerin in heparin-anticoagulated plasma and adipose tissues of WKY and SHR was determined by Western blotting. Representative blots were shown (**A**,**C**). The normalized chemerin expression relative to total protein (**B**) or total actin (t-actin) (**D**) expression was shown as relative to WKY. Data were shown as mean + SEM in bar graph ((**B**): *n* = 6, (**D**): WKY: *n* = 3, SHR: *n* = 4).

Finally, protein expression of chemerin in plasma and adipose tissues was compared between WKY and SHR (15-week-old). Protein expression of chemerin in plasma and adipose tissues in SHR did not change compared with that in WKY (Figure 6A,B: *n* = 6, Figure 6C,D: WKY: *n* = 3, SHR: *n* = 4).

## 3. Discussion

In the present study, we examined the roles of central chemerin/CMKLR1 on pathogenesis of essential hypertension, and the major findings were as follows; (1) systolic blood pressure of SHR but not WKY was decreased by CMKLR1 siRNA (i.c.v.) (Figure 2C). (2) the decrease of systolic blood pressure by CMKLR1 siRNA in SHR persisted for 3 days (Figure 3A). (3) protein expression of CMKLR1 in PVN of SHR tended to be increased compared with WKY, which was suppressed by CMKLR1 siRNA (Figure 4). (4) protein expression of chemerin in brain, peripheral plasma, and adipose tissue was not different between WKY and SHR (Figure 5 and Figure 6). Collectively, it was for the first time revealed that the increased protein expression of CMKLR1 in PVN is at least partly responsible for the systemic hypertension in SHR.

It was reported that an increased SNA in SHR was followed by an increase in systemic blood pressure. For one possible mechanism, the decreased expression of GABA receptor in PVN was proposed [20]. Moreover, it was reported that mRNA expression of angiotensin II type I receptor was increased, which mediated sympathoexcitation via the activation of N-Methyl-D-aspartate (NMDA) receptor [24]. Collectively, there are several factors contributing to the increased SNA in SHR, which lead to systemic hypertension. Recently, we have revealed that i.c.v. injection of chemerin−9 to normotensive Wistar rats induced a pressor response through the augmentation of SNA via CMKLR1 expressed in brain [23]. In the present study, protein expression of CMKLR1 in PVN of SHR tended to be increased compared with that of WKY, and CMKLR1 siRNA decreased systolic blood pressure in SHR (Figure 2 and Figure 4). Taken together, it is suggested that the increased protein expression of CMKLR1 in PVN of SHR may be partly involved in the augmentation of SNA, which is one of the causative factors for pathogenesis of essential hypertension.

It is known that an adipocytokine, leptin in peripheral blood regulates appetite through acting on central nervous system [22]. Moreover, leptin acting on central nervous system also participates in blood pressure control through the augmentation of SNA. Additionally, it was reported that expression of orexigenic genes in central nervous system was decreased by a peripheral administration with chemerin [25]. Taken together, it seems highly likely that peripheral chemerin crosses blood brain barrier and acts on central nervous system. While concentration of chemerin in plasma of the healthy human subject was approximately 50 ng/mL, that in the obese subjects that were related to hypertension through the augmentation of SNA was increased to 350 ng/mL [26,27]. Furthermore, it was reported that concentration of chemerin in plasma correlated positively with increased systemic blood pressure [28]. Thus, it was expected that chemerin, which was increased in peripheral tissue with hypertension, acted on central nervous system in SHR, and mediated pressor response through the augmentation of SNA. Unexpectedly, protein expression of chemerin in peripheral tissues (plasma and adipose tissue) in SHR did not change compared with WKY (Figure 5 and Figure 6). Thus, it seems likely that the number of CMKLR1 that can bind to chemerin in PVN was increased, leading to the augmented neuron activity and the increased systemic blood pressure.

In the present study, heart rate of WKY and SHR did not change by the i.c.v. injection of CMKLR1 siRNA. Heart rate may increase as a compensation when blood pressure decreases through the actions of SNA. However, heart rate is also controlled by parasympathetic nerve activity [29]. It is thus assumed that CMKLR1 siRNA affects not only SNA but also parasympathetic nerve activity. Then, heart rate did not change. 

It has been reported that chemerin/CMKLR1 has various functions in central nervous system (e.g., protecting neurons from an inflammation and regulating expression of genes involved in appetite) [30,31,32]. It was reported that chemerin/CMKLR1 stimulated the migration via PKC in mesenchymal stromal cell [33]. PKC is an important signal molecule in neuronal excitation [34]. PKC mediates the phosphorylation of NMDA receptor and subsequent sympathoexcitation [35,36]. Indeed, it was demonstrated that the phosphorylation of NMDA receptor was stimulated by PKC in PVN of SHR [24]. Moreover, it was reported that PKC mediated synaptic trafficking of NMDA receptor and long-term potentiation [37,38]. Therefore, it seems likely that the increased protein expression of CMKLR1 in PVN of SHR mediated the activation and/or trafficking of NMDA receptor via PKC resulting in sympathoexcitation. Additionally, it was reported that astrocyte regulates the release and uptake of neurotransmitter and participates in neuronal excitation [39]. In astrocyte, calcium influx is caused via L-type calcium channel (LTCC), which increased intracellular calcium concentration leading to a release of glutamic acid [40,41]. Since chemerin/CMKLR1 stimulates LTCC and causes calcium influx [42], it seems likely that the increased protein expression of CMKLR1 in PVN of SHR mediates the augmentation of SNA through the release of glutamic acid via the pathway activated by astrocyte.

Gut microbiome has various effects for host homeostasis contributing to blood pressure regulation [43]. For example, changes in gut microbiome in SHR reduced blood pressure through the reduction of SNA [44]. Moreover, a relationship between chemerin/CMKLR1 and gut microbiome has been reported [45]. Thus, it is possible that chemerin/CMKLR1 mediates the increased SNA through the actions on gut microbiome.

In conclusion, we for the first time revealed that the increased protein expression of CMKLR1 in PVN of SHR is at least partly responsible for an increase in systemic blood pressure. In the future, we need to explore the detailed mechanisms by which chemerin/CMKLR1 causes sympathoexcitation. Consequentially, it is expected to lead to the drug discovery targeting chemerin/CMKLR1 in central nervous system.

## 4. Materials and Methods

### 4.1. Materials

Antibody sources were as follows; anti-ChemR23 (CMKLR1; sc-398769, Santa Cruz Biotechnology, Santa Cruz, CA, USA), anti-Glyceraldehude-3-phosphate dehydrogenase (GAPDH; 016-25523, Wako, Osaka, Japan), anti-chemerin (ab112520, Abcam, Cambridge, UK), anti-total-actin (t-actin) (MAB1501; Sigma-Aldrich, St. Louis, MO, USA), and anti-mouse IgG Horseradish Peroxidase (HRP)-linked whole antibody (Cell Signaling Technology, Beverly, MA, USA).

### 4.2. Animals

Male WKY and SHR (10–17-week-old, WKY: 250–350 g, SHR: 200–320 g) (Hoshino Laboratory Animal Inc., Ibaraki, Japan) were used. Animal study was approved by the ethical committee of School of Veterinary Medicine, the Kitasato University (approval no. 19-227, 19-224), and performed in conformity with an institutional guideline of the Kitasato University.

### 4.3. Western Blotting

Western blotting was performed as described previously [23]. Briefly, the rat tissues were quickly frozen by a liquid nitrogen and crushed. They were dissolved in lysis buffer (Cell Signaling Technology) containing a protease inhibitor mixture (Nacalai Tesque, Kyoto, Japan). After centrifugation, the supernatant was obtained as protein samples. Protein concentration was determined by a bicinchoninic acid method (Pierce, Rockford, IL, USA). Equal amount of protein (10 μg) was separated by 10% sodium dodecyl sulfate-polyacrylamide gel electrophoresis (80–120 V, 1.5–2 h) and transferred (400 mV, 1.5 h) to a nitrocellulose membrane (Pall Corporation, Ann, Arbor, MI, USA). After being blocked with skim milk (0.5%), the membrane was incubated with a primary antibody (1:500 dilution, 4 °C, overnight) followed by an incubation with HRP-linked secondary antibody (1:10,000 dilution) for 45 min. Finally, the membrane was treated with chemiluminescent reagent (EZ-ECL system; Biological Industries Kibbutz, Beit-Haemek, Israel). Protein bands were visualized by an ATTO light-capture system (ATTO, Tokyo, Japan). For confirming equal loading of protein, expression of GAPDH (brain), t-actin (adipose tissue), or total protein (plasma) was examined. Expression of total protein was measured by a ponceau S staining as followings: the membrane was incubated for 5 min in a ponceau S solution containing 1% ponceau S reagent (AppliChem, Darmstadt, Germany) dissolved in 5% acetic acid solution at room temperature, and rinsed in 1% acetic acid solution. The detected bands were analyzed using a CS analyzer 3.0 software (ATTO).

### 4.4. siRNA Injection to Rats

siRNA injection (i.c.v.) was performed as described previously [23]. Briefly, WKY or SHR were anesthetized with an isoflurane (induction: 5%, maintenance: 2–3%; Wako) under a buprenorphine analgesia (50 µg/kg, s.c.; Otsuka Pharmaceutical, Tokyo, Japan). They were placed in a stereotaxic apparatus (NARISHIGE, Tokyo, Japan). The fixed head was horizontalized and siRNA was injected by a microsyringe (ITO CORPORATION, Shizuoka, Japan) at the position of 0.8 mm-anteroposterior, 1.5 mm-mediolateral from the Bregma, and 4.5 mm-dorsoventral from the surface of skull. CMKLR1 siRNA (CCAUCGUCUUCAAGUUGCA-dTdT) or Cont siRNA (0.04 nmol; Nippon Gene Material, Toyama, Japan) was mixed with an in vivo jet-PEI reagent (Polyplus transfection, Illkirch-Graffenstanden, France) and 10% glucose. The number of animals in each group was as follows; WKY (Cont siRNA, 1 day), *n* = 4; WKY (CMKLR1 siRNA, 1 day), *n* = 4; SHR (Cont siRNA, 1 day), *n* = 4; SHR (CMKLR1 siRNA, 1 day), *n* = 4; SHR (Cont siRNA, 3 days), *n* = 4; SHR (CMKLR1 siRNA, 3 days), *n* = 5.

### 4.5. Measurement of Systolic Blood Pressure and Heart Rate

To measure systolic blood pressure and heart rate in conscious condition, a tail cuff system (Softron Co. Ltd., Tokyo, Japan) was utilized as described previously [46,47]. 

### 4.6. Isolation of PVN

PVN was isolated as described previously [23]. WKY or SHR were euthanized deeply with urethane (1.5 g/kg, i.p.). The whole brain tissues were isolated. In order to separate PVN, they were cut into a slice on ice. The PVN was isolated at the position of approximately 1.0–2.0 mm-posterior from Bregma (thickness: approximately 1 mm) according to Rat Brain Atlas [48].

### 4.7. Statistics

Data were shown as mean + standard error of the mean (SEM). Statistical evaluations were performed using Mann-Whitney’s U test (Figure 1B,D, Figure 2, Figure 3, Figure 5B,D and Figure 6B,D) or one-way non-parametric test by Kruskal-Wallis method (Figure 4B). Results were considered significant when *p* value was less than 0.05.

## Data Availability

The detailed data of the current study are available from the corresponding authors upon reasonable request.

## Abbreviations

CCRL2	Chemokine (CC motif) receptor-like 2
CMKLR1	Chemokine-like receptor 1
GABA	γ-aminobutyric acid
GAPDH	Glyceraldehyde 3-phosphate dehydrogenase
GPR1	G protein-receptor 1
i.c.v.	Intracerebroventricular
LTCC	L-type calcium channel
NMDA	N-Methyl-D-aspartate
PVN	Paraventricular nucleus
PKC	Protein kinase C
RVLM	Rostral ventrolateral medulla
SHR	Spontaneously hypertensive rats
siRNA	Small interfering RNA
SNA	Sympathetic nerve activity
WKY	Wistar Kyoto rats
